# WikiPathways: Pathway Editing for the People

**DOI:** 10.1371/journal.pbio.0060184

**Published:** 2008-07-22

**Authors:** Alexander R Pico, Thomas Kelder, Martijn P van Iersel, Kristina Hanspers, Bruce R Conklin, Chris Evelo

## Abstract

WikiPathways provides a collaborative platform for creating, updating, and sharing pathway diagrams and serves as an example of content curation by the biology community.

The exponential growth of diverse types of biological data presents the research community with an unprecedented challenge and opportunity. The challenge is to stay afloat in the flood of biological data, keeping it as accessible, up-to-date, and integrated as possible. The opportunity is to cultivate new models of data curation and exchange that take advantage of direct participation by a greater portion of the community.

This combination of challenge and opportunity is especially relevant to the task of collecting biological pathway information. Pathways are critical to understanding the functions of individual genes and proteins in terms of systems and processes that contribute to normal physiology and to disease. Each biological pathway must be hewn from a mass of biological information distributed across multiple publications and databases.

The particular challenge of pathway curation is amplified, because pathways are often presented as static images that are not amenable to computation, integration, or data exchange. Furthermore, pathway experts are distributed throughout the world, and most have limited time to learn about complex databases that need their expertise. This challenge can be met by taking the opportunity to develop a new community-based model for pathway curation.

One way to engage the community is with a wiki model, as exemplified by Wikipedia [1]. We see the potential for a wiki-based pathway curation resource, coupled with an embedded graphical pathway editing tool, to meet the growing challenge presented by the influx of biological data and to provide an innovative example of content curation by the biology community (Figure 1).

**Figure 1:**
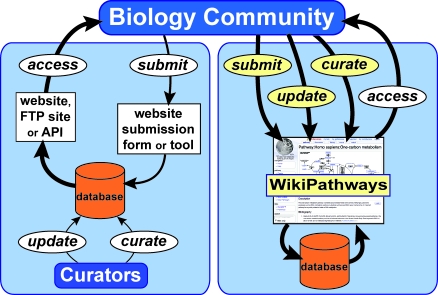
Two Models for Managing Biological Data Current biological databases provide the community with data submission forms or tools and access to the compiled data via websites, FTP sites, and, sometimes, programmatic interfaces (API). Internal curation teams organize and update the data. A wiki model for biological databases, such as WikiPathways, provides a single, intuitive interface for submitting, updating, organizing, and accessing data, allowing the community to participate in the curation process and keep up with the influx of new data. The widths of the arrows represent the relative capacity for data management in the two models.

## Facing the Challenge

The research community is experiencing massive growth in biological data, from genome and metagenome sequencing to high-throughput assays and microarray studies. This growth has created a need for models of data storage and distribution that support a continuous stream of end-user submissions, frequent updates, integrated search across databases, and access to data formats (preferably community standards) that are amenable to computational analyses. By and large, the need is being met for certain types of biological data: sequences go to GenBank and European Molecular Biology Laboratory (EMBL)-Bank, protein structures go to the Protein Data Bank (PDB), and microarray results go to Gene Expression Omnibus (GEO) and ArrayExpress. But as the influx and complexity of biological data continue to grow, so will the challenge of organizing and maintaining these databases.

Fortunately, the biology community can provide an answer that will scale with the challenge: community curation. There is a growing tendency toward information exchange that supports open access, higher-order organization, community-defined data formats, and collaborative online environments. This trend is most apparent with the growing number of open-access journals (see the Directory of Open Access Journals at http://www.doaj.org/), public databases [2], and data exchange formats [3] and ontologies [4]. To promote community curation, database maintainers must be willing to relinquish some control. After removing logistical as well as technical barriers with creative support tools, the data producer will also be the data organizer. Despite initial misgivings, such projects do succeed with the right balance of infrastructure, participation, and administrative principles, as demonstrated by Wikipedia [1], numerous open-source software projects (e.g., Linux, Apache, MySQL, Firefox), and countless scientific collaborations, including the Internet, itself. The idea of using wiki technology for biological information has been proposed in other areas, for example, genome annotation [5,6]. The EcoliWiki provides a working example of community curation focused on Escherichia coli (EcoliWiki, http://ecoliwiki.net). And WikiProteins aims to combine automated text mining with community curation to annotate biomedical concepts, including protein functions, interactions, and disease relationships [7].

## Representing Biological Pathways

Biological pathways present a special case in which the information is not directly coupled to data collection. One does not sequence or measure a pathway. Pathways comprise a myriad of interactions, reactions, and regulations, which are often identified piecemeal over extended periods and by a variety of researchers. As a result, pathway information is particularly challenging to compile and curate. Furthermore, biological pathways are often captured only as static images for publications or presentations. Consider the pathway illustrations common in textbooks and review articles that document any given field of biology. A typical signaling pathway, for example, represents receptor-binding events, protein complexes, phosphorylation reactions, translocations, and transcriptional regulation, with only a minimal set of symbols, lines, and arrows. While these simple images are powerful visual and conceptual representations, they cannot be connected to relevant biological annotations or analyzed with respect to experimental data.

A number of groups have taken on the challenge of curating and archiving biological pathways [8]. Those efforts mainly rely on internally supported teams of biologists or contracts with volunteer experts in particular fields of biology. Their curation tools typically require download, installation, and specialized training and are not designed for broad or collaborative use. Often, the barrier is simply too high for the average biologist to consider contributing their own pathway knowledge. Even when it is contributed, pathway information can remain untouched for years in the current databases, quickly becoming outdated and out of sync with the continuing stream of published discoveries. Some of us (BRC, KH, ARP) have first-hand experience with maintaining the GenMAPP [9] pathway archive in this fashion over the past 8 years. The task of submitting and updating content inevitably falls on a handful of specialists who have invested significant time installing and learning how to use the curation tools. This approach is not sustainable in the face of the growing influx of biological data. Clearly, curating all of biology is a Herculean task for any single group.

## Pathway Editing for the People

To facilitate the contribution and maintenance of pathway information by the biology community, we established WikiPathways (http://www.wikipathways.org). WikiPathways is an open, collaborative platform dedicated to the curation of biological pathways. WikiPathways thus presents a new model for pathway databases that enhances and complements ongoing efforts (see Kyoto Encyclopedia of Genes and Genomes (KEGG) at http://www.genome.jp/kegg, Pathway Commons at http://www.pathwaycommons.org/pc/, and [10]). Building on the same MediaWiki open-source software that powers Wikipedia, we added a custom graphical pathway editing tool and integrated databases covering major gene, protein, and small-molecule systems. The familiar web-based format of WikiPathways greatly reduces the barrier that prevents participation in pathway curation. More importantly, the open, public approach of WikiPathways allows for broader participation by the entire community, ranging from students to senior experts in each field. This approach also shifts the bulk of peer review, editorial curation, and maintenance to the community.

**Figure 2:**
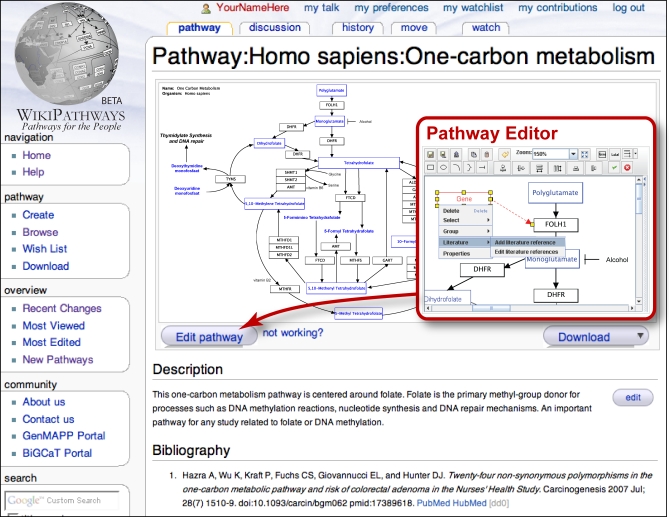
Sample WikiPathways Page Each pathway has a dedicated page for viewing and editing content. The pathway diagram is edited with an embedded applet version of PathVisio (inset). The Description and Bibliography sections can be edited in-page as well through applets that facilitate entry. Additional information about the pathway components and version history continue on the page (not shown).

Each pathway at WikiPathways has a dedicated wiki page, displaying the current diagram, description, references, version history, and component gene, protein, and metabolite lists (Figure 2). Any pathway can be edited from within its wiki page by activating an embedded pathway editor. WikiPathways uses an applet version of PathVisio—a pathway-drawing tool we developed for pathway curation (see PathVisio at http://www.pathvisio.org and unpublished data). PathVisio provides a basic palette of objects and annotations needed to represent biological processes. Gene, protein, and metabolite objects directly map to biological annotations from multiple public databases through an extensible identifier synonym database maintained at WikiPathways. The editing tool facilitates annotation with keyword search and auto-completion. Relationships between entities can easily be drawn using “smart connectors” that snap into place. Lines can even connect to other lines to intuitively represent catalysis or other mediated processes. Entities can be grouped to represent complexes and related collections of genes. The editing tool also makes it easy to annotate these entities and relationships with peer-reviewed literature references. The “Help” section of WikiPathways provides guidelines and tutorials for how to use the editor and how to best represent pathway information, as well as how to download and use the pathways in GenMAPP analyses.

After editing, an updated pathway image is displayed on the wiki page along with the version history and list of components. Users can easily monitor and undo changes, compare differences, and search for overlapping pathways. Any registered user can add a pathway to their “watch list” so that they receive email when the pathway is changed. All changes can be reversed, restoring the pathway to an earlier version. Different versions of pathways can be compared side-by-side using an integrated difference–viewing tool, customized for graphical pathway information. Using the search feature, one can locate particular pathways by name, by the genes and proteins they contain, or by the text displayed in their descriptions and comments. One can also browse the collection of pathways with combinations of species names and ontology-based categories. Currently, WikiPathways contains 544 species-specific pathways for human, mouse, rat, zebrafish, fruit fly, worm, and yeast. The mouse pathways, for example, contain 3,741 unique genes (~13% of the mouse genome). The pathway collection was nucleated with GenMAPP pathways, which were collected over the past decade from GenMAPP users. Now at WikiPathways, the collection is growing and improving through new contributions and curation, at an unprecedented rate, which we expect to dramatically increase as community participation grows.

The pathway content at WikiPathways is freely available for download in a variety of data and image formats, including GenMAPP Pathway Markup Language (GPML), which is a custom XML format that is compatible with pathway visualization and analysis tools such as Cytoscape [11], GenMAPP [9], and PathVisio (http://www.pathvisio.org). GPML allows researchers to draw and identify the molecular participants in a pathway, as well as the relationships among the participants. GPML is a work in progress, and though it does not yet have the full expressiveness of BioPAX (see the BioPAX Wiki at http://biopaxwiki.org/cgi-bin/moin.cgi) or Systems Biology Markup Language (SBML) [12], it provides the basic functionality for researchers to create appealing pathway diagrams and to perform basic statistical tests on pathways, such as overrepresentation analysis. The goal of GPML is to bridge the simple elegance of a pathway drawn on a napkin by a biologist (including its rich, human interpretability) and the growing databases of gene and protein annotations, interactions, and experimental data. We prioritized the development of GPML based on what is already available and what is most useful to the average biologist: connecting intuitive, human-readable graphics to standardized identifiers from popular databases. This allows users to accurately label entities on pathways and computationally map them to experimental data using pathway analysis software. GPML also supports the representation of relationships between entities to allow network-based visualization and analysis. In a recent “community curation event” at WikiPathways, we formalized network relationships in the human pathway archive. We plan to include a number of BioPAX elements into GPML to support data exchange, but the overriding goal for GPML is to lower the barrier for contributors of pathway information by keeping it simple. This approach resonates with the large portion of the biology community interested in basic statistical pathway analyses and figures for publications and presentations.

To assist pathway authors and curators, we are developing “bots” to survey the content and identify potential inconsistencies, redundancies, and incomplete data. The first of these bots identifies all the genes, proteins, and metabolites in any pathway that are not connected to a synonym database identifier. These reports along with additional curator tools will help contributors to submit high-quality content and make corrections where needed. We also plan to use standard biomedical ontologies to structure the content of WikiPathways and to provide organization that can scale with rapidly growing and interrelated information.

Researchers interested in particular interactions or pathways can use WikiPathways as a resource for up-to-date pathway information and as a repository for their own findings that, in turn, are immediately available in multiple data formats for analysis as well as image formats for publication. WikiPathways can be used collaboratively to create, edit, and share pathway information with any colleague who has access to a Web browser. For sensitive content that is proprietary or must first be published as an original finding, pathways can be saved locally in the GPML format, ready to be uploaded and made public at a later time. Expert curators can use WikiPathways to monitor and update pathway information associated with their fields of interest. WikiPathways is also useful to students and professors of biology, providing pathways as educational materials and the editing history of a given pathway as an example of how scientific knowledge iteratively progresses.

To encourage participation by the community we have built templates for “User pages” and “Portals.” User pages help users identify themselves and their work, whereas Portals help entire communities of users to identify themselves collectively and focus on particular pathway domains, such as diabetes-related pathways or plant pathways. By using the template, users can build a site within WikiPathways dedicated to their lab, organization, or area of interest within minutes. We are also organizing community curation events as a way to introduce new users to the curation tools and, at the same time, improve the quality of the pathway content. Future community curation events will focus on adding annotation, group representations, and literature references.

Even prior to this publication introducing WikiPathways, we have seen strong signs of community participation. Outside of the immediate group of developers, WikiPathways has already attracted ten new mouse pathways, nine new human pathways, six new zebrafish pathways, three new rat pathways, and one Portal for the micro-nutrients community. There are dozens of E. coli and plant pathways currently being converted, and three new Portals under construction. The site has over 250 registered users (10% contributing users) and has attracted developers through the Google Summer of Code program.

We envision WikiPathways being part of a broader effort to extend curation capacity to larger groups and communities. This effort does not replace current approaches involving centralized teams of curators, but rather it complements and extends them. Eventually, we would like to see wiki solutions such as WikiPathways used by current databases and curation sources. Our future directions include supporting “reference” pathways contributed by other pathway databases, and private workspaces for groups to collaboratively work on pathways before making them public. One could also imagine organizations installing local instances of WikiPathways for internal projects at research institutes or biotechnology companies. A journal, for example, could host a version of WikiPathways that only contributing authors can edit. Where the same wiki technology is used, there are opportunities for seamless integration and controlled sharing of content when it is ready to be published or released to the public site. We will continue to work toward supporting broad implementations of WikiPathways to promote contributions from established and diverse sources.

WikiPathways is an experiment. We have considerable work ahead of us in developing the GPML data model, implementing critical features and, most importantly, building a community of users and contributors. The success of WikiPathways will depend on the overall quality of its content, which will be a function of the infrastructure and administrative principles we use in addition to community participation. Features such as database connectivity, automatic consistency checks, curation tools, reversible edits, the visual difference viewer, and support by literature references will assist in tracking and reverting errant contributions, stimulating curation by the community. We anticipate that lowering the entry barrier for participation will allow for a greater capacity of curation, broader consensus on content, and ultimately, higher quality control. We are confident that WikiPathways will be a powerful resource for the research community and a vital forum for pathway curation. And we are hopeful that it will serve as an example for how the continuing flood of biological data can be managed and utilized by the community to irrigate future hypotheses and discoveries.

## Source Code

We are committed to open access and open source. All content is available under a Creative Commons License (http://creativecommons.org/licenses/by-nc-sa/3.0/). All source code for WikiPathways and the PathVisio applet is available under the Apache License, Version 2.0 (http://www.apache.org/licenses/). You can download the code from:

- http://svn.bigcat.unimaas.nl/wikipathways
- http://svn.bigcat.unimaas.nl/pathvisio

## Abbreviations

GPML: GenMAPP Pathway Markup Language
